# Clinical characteristic, treatment, and mortality among cancer and non-cancer patients presented with incidental pulmonary embolism

**DOI:** 10.3389/fmed.2025.1674173

**Published:** 2025-11-06

**Authors:** Chatree Chai-Adisaksopha, Warawut Chaiwong, Piangrawee Niprapan, Adisak Tantiworawit, Chaiwat Bumroongkit

**Affiliations:** 1 Division of Hematology, Department of Internal Medicine, Chiang Mai University, Chiang Mai, Thailand; 2 Division of Pulmonary, Critical Care and Allergy, Department of Internal Medicine, Chiang Mai University, Chiang Mai, Thailand

**Keywords:** incidental pulmonary embolism, suspected pulmonaryembolism, pulmonary embolism, PE, cancer associated thrombosis

## Abstract

**Introduction:**

Cancer patients are at an increased risk of developing venous thrombosis Advances in multidetector computed tomography (CT) scanners have facilitated the detection of pulmonary embolism (PE). However, the natural course of incidental PE (IPE), particularly in cancer patients, remains controversial.

**Methods:**

This retrospective cohort study was conducted at a tertiary medical center in Thailand. Patients aged 15 years or older who were diagnosed with PE between 2011 and 2020 were included. The study population was divided into two groups: the IPE group and the suspected PE (SPE) group. The primary outcome was 30-day mortality.

**Results:**

A total of 736 patients with acute PE were included in the analysis, with 281 classified as having IPE and 455 as having SPE. Active cancer was more prevalent in the IPE group compared to the SPE group (70.8% vs. 46.6%, *p* < 0.001). IPE presented with fewer PE-related symptoms and lower markers of severity, and received LMWH more often but with longer time to first anticoagulant (median 24 h vs. 2.78 h; *p* < 0.001). Thirty-day mortality was observed in 25.72% of the IPE group and 30.24% of the SPE group (*p* = 0.064). Subgroup analysis of cancer patients showed that those with IPE had a lower mortality rate (24.12%) compared to those with SPE (44.34%, *p* < 0.001). However, in adjusted Cox models, IPE was not independently associated with 30-day mortality (HR 1.42, 95% CI 0.84–2.43, *p* = 0.194).

**Conclusion:**

IPE is common in cancer and is associated with delayed anticoagulation but similar adjusted short-term mortality to SPE. System-level pathways to expedite treatment for IPE may improve care, especially in cancer patients.

## 1 Introduction

Venous thromboembolism (VTE) comprises of deep vein thrombosis (DVT) and pulmonary embolism (PE) is common complications in cancer patients. The incidence of VTE among cancer patients were found to be 4–7 times higher than normal population (1).

Incidental pulmonary embolism (IPE), identified during imaging conducted for reasons other than suspected VTE (SPE), has become increasingly recognized. The prevalence of IPE is reported in approximately 1–2.6% of chest CT scans (2, 3). In cancer patients, however, IPE is detected more frequently, with rates ranging from 3.3 to 6.4% (4–6).

Despite a lack of overt symptoms. IPE presents unique clinical challenges due to its frequent asymptomatic nature and the potential for serious complications, which may go untreated without proper detection.

The prognosis of IPE relative to SPE remains controversial. Several studies reported comparable prognosis for IPE and SPE in patients with cancer (7, 8), however, some studies reported that patients with IPE had lower mortality rate than those with SPE (9, 10).

The objectives of this study were to compare clinical characteristics, treatment patterns (including time-to-anticoagulation), and short-term mortality between IPE and SPE, overall and stratified by cancer status.

## 2 Methods

### 2.1 Study design and setting

This retrospective study was conducted at Chiang Mai University, a tertiary care center in Chiang Mai, Thailand, between January 2011 and December 2020 (11, 12). We identified all potential cases by the International Classification of Diseases, Tenth Revision (ICD-10) codes across the entire hospital admission databases. Patients aged 15 years or older who were admitted to the hospital with acute pulmonary embolism (PE) were included based on the ICD-10 codes: I26.0 (pulmonary embolism with acute cor pulmonale) and I26.9 (pulmonary embolism without acute cor pulmonale). Investigators, together with radiologists, reviewed the PE diagnoses of included patients according to the following criteria: evidence of thrombus in the pulmonary artery or its branches on computed tomography pulmonary angiography, or thrombus presence in the pulmonary artery or its branches on chest CT with contrast (12). Patients were excluded from the study if they had missing data for critical values needed to calculate simplified Pulmonary Embolism Severity Index (sPESI) risk score, if they were duplicate cases, non-Asian patients, or if they had chronic thromboembolic pulmonary hypertension. The study was approved by the Research Ethics Committee of the Faculty of Medicine, Chiang Mai University, Thailand (approval number: 329/2566).

We classified PE into two mutually exclusive categories. IPE was defined as thrombus identified on a CT performed for an indication other than suspected PE (e.g., cancer staging, treatment response, surveillance, or evaluation of non-cardiopulmonary conditions), with no documented clinical suspicion for PE at the time of ordering. SPE was defined as PE diagnosed on imaging obtained because PE was suspected clinically, prompted by ≥1 of the following: acute dyspnea, pleuritic chest pain, syncope, hemoptysis, tachycardia, hypoxemia, or hemodynamic instability.

### 2.2 Data collection

Patients’ demographic data, including age, sex, and comorbid diseases (hypertension, diabetes, obesity, congestive heart failure, obstructive sleep apnea, and chronic obstructive pulmonary disease), as well as signs and symptoms of PE, were collected. PE-related investigations, including electrocardiography, right ventricular dysfunction (determined by either echocardiography or CT scan), troponin T levels, and D-dimer levels, were also recorded. Patients with a history of active cancer within 6 months prior to the PE diagnosis were classified as cancer patients; otherwise, they were classified as non-cancer patients. Cancer stage and the Eastern Cooperative Oncology Group (ECOG) scale were also retrieved. The PESI score was calculated for each patient. Data on PE treatment, including the type of anticoagulant used and the time from PE diagnosis to the first anticoagulant administration, were collected. Death was identified through the medical records, with the date of death recorded. For patients with no documentation of death in the medical records, investigators cross-checked with government databases to obtain the current status (alive or deceased).

### 2.3 Statistical analyses

The primary outcomes of the study were the 1-month and 3-month mortality rates of patients with IPE, compared with those with SPE. Subgroup analysis was performed based on the cancer status of the patients.

Descriptive analysis was used for demographic data. Categorical data were presented as numbers and percentages, while continuous data were presented as the mean with standard deviation (SD) or median with interquartile range (IQR), as appropriate. Differences between categorical data were tested using either the Chi-square test or Fisher’s exact test. Differences between continuous data were tested using either Student’s *t*-test or the Mann–Whitney U test for normal distribution and non-normal distribution, respectively.

Logistic regression and Cox proportional hazards models were used to determine the association between the type of PE (IPE versus SPE) and mortality. A multivariable analysis was performed for 30-day mortality in cancer patients. Covariates for the multivariable Cox proportional hazards model were chosen *a priori* based on clinical relevance and variables showing significant association in univariable analyses (*p* < 0.10). The proportional hazards (PH) assumption was evaluated using Schoenfeld residuals and by visual inspection of log-minus-log survival curves. When evidence of PH violation was detected, we performed a sensitivity analysis using a Weibull parametric survival model, which accommodates non-proportional hazards. The Weibull model produced estimates consistent with those of the Cox model, confirming the robustness of our results. All analyses were conducted on a complete-case basis. Variables with >5% missingness that were not incorporated in the regression models. The *p*-value of <0.05 was considered statistically significant. All statistical analyses were conducted using STATA version 15 (StataCorp, College Station, TX, United States).

## 3 Results

### 3.1 Baseline characteristics and initial clinical presentation

Of 736 included patients, 281 had IPE and 455 had SPE. Baseline clinical characteristics of the two patient groups are shown in Table 1. Males accounted for 45.5% in the IPE group and 40.4% in the SPE group, with a mean age of 58.8 ± 14.27 years in the IPE group and 57.52 ± 16.16 years in the SPE group. The proportion of patients with comorbid diseases was comparable between groups, except for a lower percentage of patients with a history of congestive heart failure in the IPE group compared to the SPE group (1.42% vs. 8.37%, respectively). There was a significantly higher proportion of patients with active cancer in the IPE group (70.8%) compared to the SPE group (46.6%).

**Table 1:** Baseline characteristics.

Characteristics	Incidental PE	Suspected PE	*P*-value
	(*n* = 281)	(*n* = 455)	
Age mean (SD)	58.80 ± 14.27	57.52 ± 16.16	0.274
Male, n (%)	128 (45.55)	184 (40.44)	0.173
Co-morbid disease, n (%)			
Hypertension	113 (40.21)	204 (44.84)	0.219
Diabetes	42 (14.95)	74 (16.26)	0.634
Obesity	2 (0.71)	10 (2.20)	0.145
Heart failure	4 (1.42)	38 (8.37)	<0.001
OSA	5 (1.78)	10 (2.20)	0.696
COPD	11 (3.91)	31 (6.83)	0.098
Cancer, n (%)			<0.001
Yes	199 (70.8)	212 (46.6)	
No	82 (29.2)	243 (53.4)	
Cancer stage, n (%)			0.76
I-III	110 (55.28)	98 (46.23)	
IV	89 (44.72)	114 (53.77)	
ECOG, n (%)			0.005
0–1	68 (34.17)	46 (21.70)	
>1	131 (65.83)	166 (78.30)	
Signs and symptoms			
Dyspnea	40 (14.23)	231 (50.77)	<0.001
Chest pain	12 (4.29)	101 (22.25)	<0.001
Tachycardia (HR > 110)	73 (25.98)	268 (59.03)	<0.001
Tachypnea	14 (4.98)	146 (32.09)	<0.001
Systolic BP	120.69 ± 17.06	114.04 ± 23.94	<0.001
Diastolic BP	74.49 ± 11.08	70.93 ± 15.30	<0.001
Shock (BP < 90/60)	3 (1.07)	46 (10.11)	<0.001
SpO_2_ < 90%	25 (8.99)	312 (69.18)	<0.001
Investigations			
Sinus tachycardia	86(30.60)	318 (69.89)	<0.001
S1Q3T3	12 (4.27)	144 (31.65)	<0.001
RV dysfunction	2 (2.99)	127 (47.57)	<0.001
RV/LV > 1	12 (4.33)	175 (38.46)	<0.001
Trop-T level	44.41 ± 61.29	149.94 ± 275.20	<0.001
D-dimer level	18999.29 ± 30387.04	17209.15 ± 22618.52	0.5718
Site of PE			
Central	79 (28.11)	236 (51.82)	<0.001
Lobar	46 (16.37)	152 (33.41)	<0.001
Segmental	221 (78.11)	382 (83.96)	0.069
Subsegmental	46 (16.37)	70 (15.36)	0.705
sPESI score			
Low-risk	36 (15.13)	27 (6.01)	<0.001
High-risk	202 (84.87)	422 (93.99)	

SD, standard deviation; PE, pulmonary embolism; n, number; OSA, obstructive sleep apnea; COPD, chronic obstructive pulmonary disease; ECOG, the Eastern Cooperative Oncology Group score; HR, heart rate; BP, blood pressure; SpO_2_, saturation of peripheral oxygen; sPESI, simplified Pulmonary Embolism Severity Index.

Patients with IPE had a significantly lower proportion of PE-specific signs and symptoms, including dyspnea, chest pain, tachycardia, tachypnea, shock, and low peripheral oxygen saturation. Similarly, patients with IPE had a significantly lower proportion of PE-specific findings, including sinus tachycardia, S1Q3T3 signs on electrocardiography, and right ventricular dysfunction. The mean troponin T level was significantly lower in the IPE group compared to the SPE group (44.41 ± 61.29 vs. 149.94 ± 275.20). With respect to the site of PE, patients with IPE had a lower proportion of central or lobar PE compared to those with SPE. Additionally, fewer patients in the IPE group were classified as high-risk according to the sPESI score (84.87%) compared to those in the SPE group (93.99%).

### 3.2 Treatment of pulmonary embolism

Table 2 demonstrates the type of anticoagulant used and the time from PE diagnosis to first anticoagulant treatment. A higher proportion of patients in the IPE group received a prescription for low-molecular-weight heparin (LMWH) (84.23%) compared to those in the SPE group (73.33%), with *p* < 0.001.

**Table 2:** Treatment of pulmonary embolism.

Characteristics	Incidental PE (*n* = 241)	Suspected PE (*n* = 420)	*P*-value
Type of anticoagulant, n (%)			
Warfarin	11 (4.56)	29 (6.90)	0.241
LMWH	203 (84.23)	308 (73.33)	0.001
DOAC	6 (2.49)	3 (0.71)	0.080
Time from PE diagnosis to first anticoagulant treatment (hours)			
Median (IQR)	24 (109.03)	2.78 (14.76)	<0.001
Cancer			
Median (IQR)	31.25 (120.21)	2.73 (15.31)	<0.001
Non-cancer			
Median (IQR)	11.55 (96.83)	2.86 (14.58)	<0.001

PE, pulmonary embolism; LMWH, low-molecular-weight heparin; DOAC, direct oral anticoagulant; IQR, interquartile range.

The median time from PE diagnosis to initiation of anticoagulant therapy was significantly longer in patients with incidental IPE compared with those with SPE (median 24.0 h [IQR 109.3] vs. 2.78 h [IQR 14.76]; *p* < 0.001). This delay persisted when patients were stratified by cancer status: both cancer and non-cancer patients with IPE received anticoagulation later than their counterparts with SPE (Table 2). Among patients with IPE, those with cancer experienced an even longer delay to anticoagulation (median 31.25 h [IQR 120.21]) compared with non-cancer patients (median 11.55 h [IQR 96.83]; *p* < 0.001).

### 3.3 All-cause mortality

**Figure 1:**
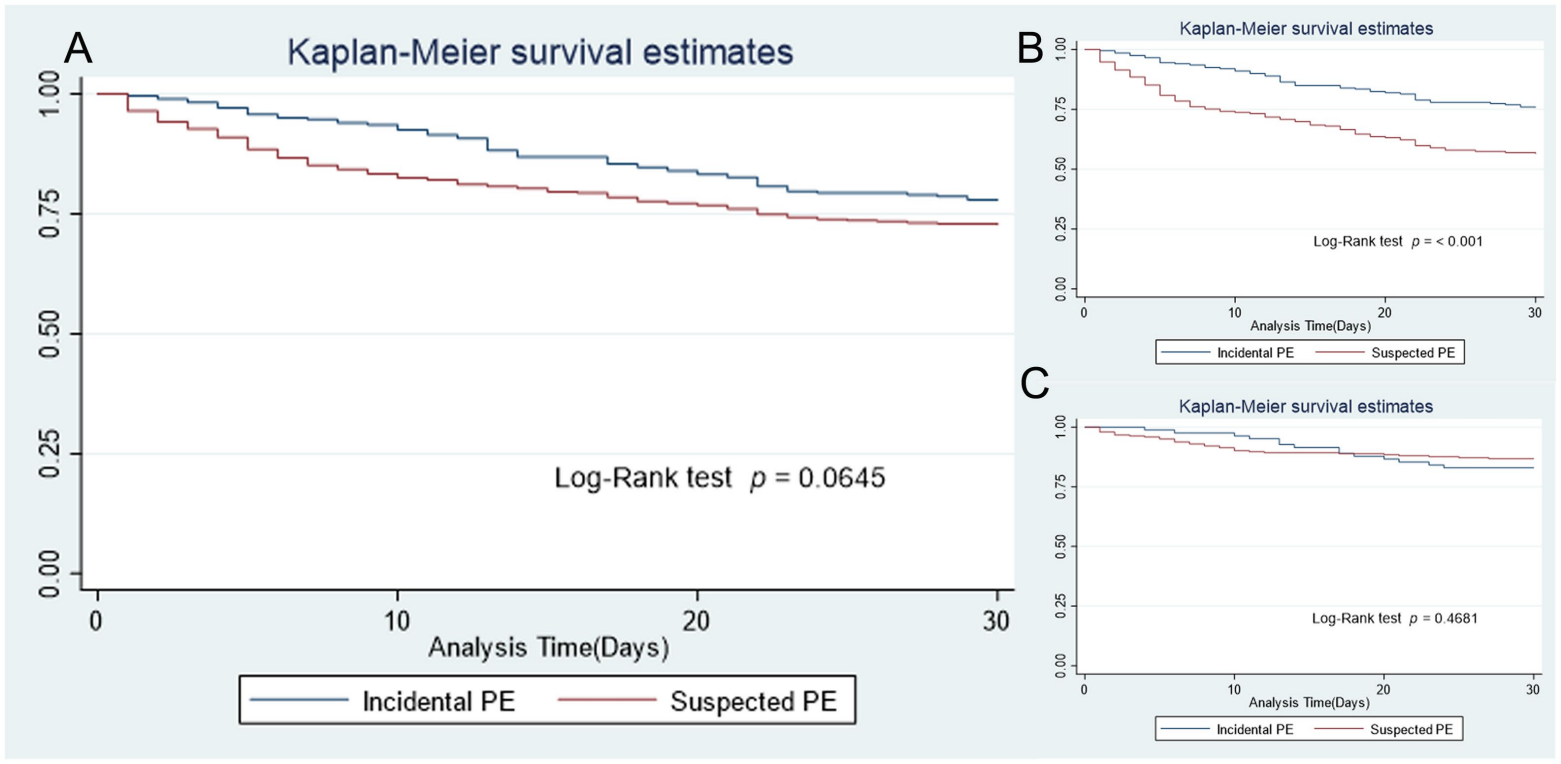
Overall survival between incidental PE and suspected PE: **(A)** all cases, **(B)** cancer patients, **(C)** non-cancer patients.

Table 3 shows the 30-day and 90-day mortality of patients, categorized by IPE versus SPE and cancer versus non-cancer status. Thirty-day mortality was observed in 25.72% of patients in the IPE group and 30.24% in the SPE group. The Kaplan–Meier curve shows no significant difference in the risk of all-cause mortality between patients in the IPE and SPE groups (log-rank *p* = 0.064; Figure 1A).

**Table 3:** All-cause mortality of patients with pulmonary embolism.

Diagnosis	Death at 30 days			Death at 90 days		
	Incidental PE, n (%)	Suspected PE, n (%)	*P*-value	Incidental PE, n (%)	Suspected PE, n (%)	*P*-value
Total patients	62 (25.72)	127 (30.24)	0.252	116 (48.13)	186 (44.28)	0.382
Cancer	48 (24.12)	94 (44.34)	<0.001	93 (46.73)	13 (63.68)	<0.001
Non-cancer	14 (17.07)	33 (13.58)	0.195	23 (28.05)	51 (20.99)	0.004

PE, pulmonary embolism.

Subgroup analysis of cancer patients revealed that those with IPE had a lower risk of death (24.12%) compared to those in the SPE group (44.34%), *p* < 0.001 (Table 2 and Figure 1B). In contrast, analysis of non-cancer patients did not show a significant difference in mortality between the IPE group (17.07%) and the SPE group (13.58%) (Table 2 and Figure 1C).

Ninety-day all-cause mortality was observed in 48.13% of patients in the IPE group and 44.28% in the SPE group (*p* = 0.382). Among cancer patients, those in the SPE group had a higher risk of death compared to those in the IPE group (63.68% vs. 46.73%, *p* < 0.001). In contrast, among non-cancer patients, those with IPE had a higher risk of death (28.05%) compared to those with SPE (20.99%), with *p* = 0.004.

### 3.4 Univariable and multivariable analysis

Table 4 presents the Cox proportional hazards model for evaluating risk factors associated with all-cause mortality at 30 days. The univariable analysis indicates that patients with IPE had a lower risk of all-cause mortality compared to those with SPE (HR 0.47, 95% CI 0.33–0.67, *p* < 0.001). However, after adjusting for potential confounders (dyspnea, tachycardia, systolic blood pressure, desaturation, ECOG performance status, and sPESI), no significant association was found between IPE and all-cause mortality (HR 1.42, 95% CI 0.84–2.43, *p* = 0.194). We identified the risk factors of 30-day mortality that were associated with higher risk of 30-day mortality, including dyspnea, tachycardia, desaturation and ECOG performance status greater than 1.

**Table 4:** Univariable/multivariable analysis of 30-day all-cause mortality.

Univariable			Multivariable		
Factors	HR (95%CI)	*P*-value	Factors	Adjusted HR (95%CI)	*P*-value
Age	0.99(0.98–1.01)	0.485			
PE			PE		
Suspected PE	Reference	<0.001	Suspected PE	Reference	0.194
Incidental PE	0.47(0.33–0.67)		Incidental PE	1.42(0.84–2.43)	
Dyspnea	2.41(1.60–3.65)	<0.001	Dyspnea	1.95(1.13–3.37)	0.016
Tachycardia	2.42(1.71–3.41)	<0.001	Tachycardia	1.66(1.13–2.45)	0.010
SBP	0.99(0.98–1.00)	0.114			
SpO_2_ < 90	2.54(1.81–3.59)	<0.001	SpO_2_ < 90	1.67(1.06–2.65)	0.028
RV/LV>1	1.49(0.98–2.29)	0.065			
ECOG			ECOG		
0–1	Reference	<0.001	0–1	Reference	0.005
>1	2.55(1.60–4.05)		>1	1.98(1.22–3.21)	
sPESI					
Low-risk	Reference	0.404			
High-risk	2.31(0.32–16.53)				

PE, pulmonary embolism; HR, hazard ratio; CI, confidence interval; SBP, systolic blood pressure; RV, right ventricle; LV, left ventricle; ECOG, the Eastern Cooperative Oncology Group score; sPESI, simplified Pulmonary Embolism Severity Index.

We assessed the PH assumption for each covariate and for the overall model. Minor deviations from proportionality were noted; therefore, a supplementary Weibull survival model was fitted as a sensitivity analysis. The hazard ratio for IPE versus SPE from this model was comparable to that obtained from the Cox model, indicating that the violation did not materially affect our findings.

## 4 Discussion

The incidence of IPE in cancer patients undergoing baseline or follow-up CT scan was found to be 3% per year (13). Similarly, a systematic review of 12 studies, involving 28,626 patients reported that the incidence of IPE in cancer patients was 3.36% (95% CI 3.15–3.57) (9). IPE was frequently found during the first year after cancer diagnosis (14). The presence of IPE had a negative impact on the outcomes of cancer patients, with those diagnosed with IPE having a higher risk of death compared to those without PE (14, 15). However, the management and outcome of IPE remain controversial.

This study aimed to compare the clinical presentation, laboratory findings, and sites of PE in patients with incidentally detected IPE versus those with SPE. Additionally, we compared clinical outcomes, specifically all-cause mortality, by categorizing patients based on their cancer and non-cancer status.

The major findings of this study include that patients with IPE had a significantly lower proportion of signs and symptoms related to acute PE, such as dyspnea, chest pain, or desaturation. Consequently, indicators of more severe PE, such as right ventricular dysfunction or elevated troponin T levels, were less commonly observed in patients with IPE compared to those with SPE. Additionally, patients with SPE were more likely to have a proximal (central and lobar) thrombus and to be classified as having high-risk PE according to the sPESI score.

It is not surprising that IPE was associated with fewer PE related signs and symptoms. Wang et al. conducted a retrospective study of 180 cancer patients with PE (88 with IPE and 92 with SPE) (10) and found that patients with IPE had less frequent hemoptysis, dyspnea, cough, fatigue and chest pain (10). In contrast to the present study, Want et al. reported that IPE patients had higher incidence of main or lobar thrombus (62.5%), compared to SPE (10.9%) (10). Additionally, Lu et al. found that patients with IPE had a numerically lower proportion of proximal PE (33.0%) compared to those with SPE (40.0%) (16).

We observed that patients with IPE were more likely to receive LMWH compared to those with SPE. This finding may be attributed to the higher proportion of patients with active cancer in the IPE group (70.8%) versus the SPE group (46.6%) at the time of diagnosis. More importantly, the median time to receive the first dose of anticoagulant was significantly longer among IPE patients compared to SPE patients (24 h versus 2.8 h). This delay was even more pronounced among cancer patients with IPE, who had a median time of 33.25 h to receive anticoagulation, compared to 11.55 h for non-cancer patients with IPE.

The longer time to initiate anticoagulation may be due to the clinical service structure. Cancer patients, particularly those undergoing scheduled CT scans for baseline or follow-up assessments, often have a subsequent clinic visit 2–4 weeks later to review their CT scan results. During this visit, treating physicians would discover the presence of PE and prescribe anticoagulation. In contrast, patients undergoing CT scans due to suspected PE receive anticoagulant treatment immediately after the physicians receive the report, resulting in shorter initiation times.

The all-cause mortality at 30-day of patients in the present study was comparable between patients with IPE (25.72%) and those with SPE (30.24%). Subgroup analysis revealed that cancer patients with IPE had a higher likelihood of death compared to those with SPE. However, no significant differences in mortality were observed among non-cancer patients. After adjusting for potential confounders, there was no significant difference in terms of association between IPE and 30-day mortality in cancer patients.

The association between IPE and mortality in cancer patients remains controversial. A retrospective study of 509 Chinese cancer patients with either IPE or SPE found a slightly lower mortality rate at 6 months among patients with IPE (17.8%) compared to those with SPE (23.3%), with a log-rank *p*-value of 0.214 (16). The study also identified different risk factors associated with an increased risk of 30-day mortality, including lung/pleura cancers, upper gastrointestinal cancers, and bilateral PE (16).

An analysis of a prospective cohort study from Mayo Clinic Rochester, which included 562 IPE and 855 SPE patients, found that patients with IPE had a higher mortality rate (46.45%) compared to those with SPE (23.47%) (17). However, after adjusting for age, antiplatelet therapy, metastases, and cancer location, there was no statistically significant association between IPE and mortality (17). The numerically higher mortality in IPE patients in this study may be explained by the fact that IPE was more common among cancer patients, who typically have a higher incidence of metastases and certain cancer locations.

In addition, another finding from the Mayo Clinic Rochester cohort was that patients without cancer had higher mortality rate in IPE group as compared to SPE group (15.95% versus 7.18%, respectively) (17). The higher incidence of mortality among non-cancer patients may be associated with underlying conditions, such as cardiovascular disease, a history of cancer, non-specific symptoms, or major multiple traumas (17). This finding was consistent with the present study, which demonstrated a higher incidence of 30-day mortality in the IPE group compared to the SPE group (17.07% vs. 13.58%), as well as 90-day mortality (28.05% vs. 20.99%).

In contrast, a single-center, retrospective study from China, which included 180 cancer patients (88 with IPE and 92 with SPE), reported that patients with IPE had better overall survival at both 30 days and 90 days (median 314.5 days) compared to those with SPE (median 192.0 days) (10). After adjusting for potential confounders, patients with SPE were found to have an increased risk of mortality, with HR of 1.82 (95% CI, 1.10–2.42) (10).

The inconsistency of findings regarding the association between the type of PE (IPE versus SPE) and mortality outcomes across various studies may be explained by the heterogeneous populations, including factors such as cancer stage and site, co-morbid conditions, treatment regimens for the primary cancer, anticoagulant management, and study design.

The major clinical implication of this study concerns the delay in anticoagulant initiation among patients with IPE, particularly those with cancer. In our cohort, patients with IPE experienced a markedly longer time to treatment compared with those with SPE (median 24 h vs. 2.78 h). Among cancer patients with IPE, the delay was even more pronounced (median 31.25 h) compared with non-cancer IPE patients (median 11.55 h). Although IPE status was not independently associated with 30-day mortality after adjustment, this treatment delay remains clinically significant, as early anticoagulation is the standard of care and is known to reduce early mortality and recurrence risk. These findings highlight a systems-level gap in the management of IPE. Establishing rapid notification and referral pathways for incidentally detected PE could help expedite treatment initiation and improve care consistency across institutions.

The strengths of the present study include the inclusion of a homogenous group of Asian patients, allowing us to better understand the natural course of disease in patients with IPE or SPE, which may differ from that in other racial groups. Secondly, we demonstrate the real-world setting of patients with PE, identifying a crucial issue in IPE patients who received significantly delayed treatment. This finding may be relevant to other medical centers where no specific protocol exists to alert healthcare providers to initiate anticoagulant treatment in such patients. Thirdly, we ensured the accuracy of the mortality outcome by reviewing medical records and cross-checking with population databases to verify that the mortality data was precise.

However, there are some limitations in the present study. Firstly, we captured all-cause rather than cause-specific mortality; we therefore cannot separate deaths from cancer progression, recurrent VTE, or bleeding, which could bias effect estimates toward the null if competing risks differ between IPE and SPE. Secondly, we did not systematically collect recurrent VTE or major bleeding during follow-up, precluding a net clinical benefit analysis of anticoagulation timing. Thirdly, our cohort comprised exclusively Asian patients treated within a single tertiary center; differences in cancer epidemiology, comorbidity profiles, and care pathways may limit generalizability to non-Asian settings. Lastly, information on long-term anticoagulant therapy, including the specific agents and dosages used, was not collected because the study was designed to evaluate short-term outcomes in patients with PE. These limitations should be considered when applying our findings to decision-making; they also highlight targets for future prospective studies incorporating rapid-treatment protocols and adjudicated cause-specific outcomes.

## 5 Conclusion

IPE was more commonly found among cancer patients and was associated with less severe signs and symptoms related to PE compared to SPE. Patients with IPE experienced significant delays in receiving anticoagulant treatment. There were no significant differences in 30-day and 90-day mortality between patients with IPE and those with SPE. Patients diagnosed with IPE should be treated in the same manner as those with SPE.

## Data Availability

The original contributions presented in the study are included in the article/supplementary material, further inquiries can be directed to the corresponding author.
